# Comparative Toxicity of Fly Ash: An In Vitro Study

**DOI:** 10.3390/molecules26071926

**Published:** 2021-03-30

**Authors:** Elvira Rozhina, Ilnur Ishmukhametov, Läysän Nigamatzyanova, Farida Akhatova, Svetlana Batasheva, Sergey Taskaev, Carlos Montes, Yuri Lvov, Rawil Fakhrullin

**Affiliations:** 1 Bionanotechnology Lab, Institute of Fundamental Medicine and Biology, Kazan Federal University, Kreml Uramı 18, 420008 Kazan, Republic of Tatarstan, Russia; rozhinaelvira@gmail.com (E.R.); irishmukhametov@gmail.com (I.I.); lyaysan.nigamatzyanova@gmail.com (L.N.); akhatovaf@gmail.com (F.A.); svbatasheva@gmail.com (S.B.); 2 Physics Department, Chelyabinsk State University, 129 Bratiev Kashirinykh St., 454001 Chelyabinsk, Russia; s.v.taskaev@gmail.com; 3 Institute for Micromanufacturing, Louisiana Tech University, Ruston, LA 71272, USA; montecarlostar@hotmail.com (C.M.); ylvov@coes.latech.edu (Y.L.)

**Keywords:** fly ash toxicity, airborne microparticles, hyperspectral microscopy, cell viability, DNA comet assay

## Abstract

Fly ash produced during coal combustion is one of the major sources of air and water pollution, but the data on the impact of micrometer-size fly ash particles on human cells is still incomplete. Fly ash samples were collected from several electric power stations in the United States (Rockdale, TX; Dolet Hill, Mansfield, LA; Rockport, IN; Muskogee, OK) and from a metallurgic plant located in the Russian Federation (Chelyabinsk Electro-Metallurgical Works OJSC). The particles were characterized using dynamic light scattering, atomic force, and hyperspectral microscopy. According to chemical composition, the fly ash studied was ferro-alumino-silicate mineral containing substantial quantities of Ca, Mg, and a negligible concentration of K, Na, Mn, and Sr. The toxicity of the fly ash microparticles was assessed in vitro using HeLa cells (human cervical cancer cells) and Jurkat cells (immortalized human T lymphocytes). Incubation of cells with different concentrations of fly ash resulted in a dose-dependent decrease in cell viability for all fly ash variants. The most prominent cytotoxic effect in HeLa cells was produced by the ash particles from Rockdale, while the least was produced by the fly ash from Chelyabinsk. In Jurkat cells, the lowest toxicity was observed for fly ash collected from Rockport, Dolet Hill and Muscogee plants. The fly ash from Rockdale and Chelyabinsk induced DNA damage in HeLa cells, as revealed by the single cell electrophoresis, and disrupted the normal nuclear morphology. The interaction of fly ash microparticles of different origins with cells was visualized using dark-field microscopy and hyperspectral imaging. The size of ash particles appeared to be an important determinant of their toxicity, and the smallest fly ash particles from Chelyabinsk turned out to be the most cytotoxic to Jukart cells and the most genotoxic to HeLa cells.

## 1. Introduction

Coal-consuming industries are among the main sources of air pollution with fly ash [1,2,3]. The content of solid microparticles in the air in an urban environment can exceed 500 μg/m^3^, while in rural or remote areas, it is usually less than 5 μg/m^3^ [4,5]. For intensively operating electric power and metallurgical plants, fly ash mainly consists of aluminosilicates with minor admixtures of other oxides and carbon. The International Agency for Research on Cancer (IARC) classified such micro/nanoparticles and ambient air pollution as carcinogenic to humans [6]. In addition to the direct negative effect of airborne fly ash solid particles at the place of origin, a significant danger lies in their transportation with air currents over long distances. For example, dust and fly ash from Asia can travel thousands of kilometers downwind over eastern coastal China [7], Korea [8], Japan [9], and the North Pacific [10], and it even arrives in the US [11], significantly affecting these regions. Even in developed countries, air pollution control has not yet adequately reduced their concentration in urban areas, especially in the vicinity of large coal electric power stations and metallurgic plants [12]. There are insufficient data on the effects of different air dust types, especially micrometer-size fly ash, on human cells. The fly ash generated because of coal combustion, despite being collected and utilized [13], remains in significant quantities in ash ponds and landfills, acting as a significant contributor to air pollution [14]. The contact of fly ash with water surface in seas, lakes, and rivers is one of the main ways to accumulate it from the air, causing a long-lasting environmental hazard [13]. In addition, the concentrations of trace metals in fly ash are four to 10 times higher than in coal [15].

Large disasters have occurred, such as the 2008 Kingston Fossil Plant coal fly ash slurry spill, in which 4.2 million cubic meters of coal fly ash slurry were released into a large residential area in the state of Tennessee. Although no deaths were reported from the initial spill, at least 30 employees involved in the clean-up have reportedly died due to illnesses including brain cancer, lung cancer, and leukemia as a result of exposure to the toxic coal ash [16]. A 2010 report published by Physicians for Social Responsibility profiled 10 cases in which fly ash impacted communities’ health across the United States [17]. In 2015, the EPA published a Coal Combustion Residuals (CCR) regulation, which, although still classifying coal fly ash as non-hazardous, places new restrictions on landfills and surface impoundments as an attempt to prevent the leaking of toxic metals to groundwater [18].

Moreover, various practical applications are currently proposed for fly ash, such as the production of new insulating materials, synthetic geopolymers, and carbon nanomaterials for electronics [19], as well as using it as a source of nutrients for plant growth [20]. However, before the wide-ranging practical use of fly ash would be possible, the accumulation of reliable information on the fly ash toxicity and genotoxicity is required.

The physicochemical and cytotoxic properties of fly ash of various origins are actively studied [21,22,23]. The harmful effects of coal fly ash were previously attributed to the presence of trace elements [24] as well as the particle size distribution of the samples [25,26]. Here, we analyzed the alumosilicate fly ash microparticles collected from several electric power stations and a metallurgic plant and assessed their toxicity in vitro using the HeLa cells (human cervical cancer cells) and Jurkat cells (immortalized human T lymphocytes), typical models in numerous cell culture-based experiments. The fly ash samples were collected in the USA and Russian Federation industrial centers. The particles were characterized using atomic force microscopy and dynamic light scattering; the localization of fly ash microparticles in human cervical cells was shown using dark-field microscopy supplemented with hyperspectral imaging [27]. The cytotoxicity and genotoxicity of the investigated solid particles were evaluated as moderate at concentrations below 10 mg/mL (i.e., for ponds, it is ≈10,000 g/m^3^) and it was higher for fly ash particles sized less than 1 µm and concentration of 20 mg/mL.

## 2. Results and Discussion

### 2.1. Characteristics of Coal Fly Ash Particles

The hydrodynamic diameters (mm) and ζ-potentials of fly-ash particles dispersed in distilled water at 25 °C were obtained with laser light scattering (Table 1).

Analysis of the particle size distribution suggested that the sample from Chelyabinsk was best dispersed in water. All samples were negatively charged with ζ-potentials around −30 mV providing relatively good colloidal stability in water, except for the Dolet Hills specimen, which was less charged according to the measured ζ-potentials. Negative zeta potential on fly ash is related to an excess of SiO_2_ on the particle surface (for example, pure silica particles have a zeta potential of ca −60 mV).

In terms of chemical composition, the fly ash studied is ferro-alumino-silicate mineral containing substantial quantities of Ca and Mg, and a negligible concentration of K, Na, Mn, and Sr, which corresponds to the composition of fly ash described in the works of other authors [28]. Table 2 presents the elemental composition of fly ash samples collected from different power plants. Fly ash particles demonstrated differences in chemical composition between samples and allowed classifying them into two groups according to the standard specification ASTM C618 [29].

The major and minor ash-forming elements in fly ash were determined using an easy and low-cost way [30]. The samples from Rockdale, TX and Dolet Hills, LA with low content of Ca, and with a total content of SiO_2_ + Al_2_O_3_ + Fe_2_O_3_ higher than 70% correspond to the silica-rich class F, which is usually formed by the combustion of bituminous coal or anthracite [31,32]. At the same time, the samples from Rockport, IN and Muskogee, OK with lime content greater than 15% and the total content of SiO_2_ + Al_2_O_3_ + Fe_2_O_3_ between 50% and 70% correspond to the calcium-rich class C, which is typically produced from lignite or sub-bituminous coal combustion [31]. The EDS analysis revealed amounts of S, Mg, and Na in fly ash samples and consistent with the results of the XRD (Table S1). 

**Figure 1:**
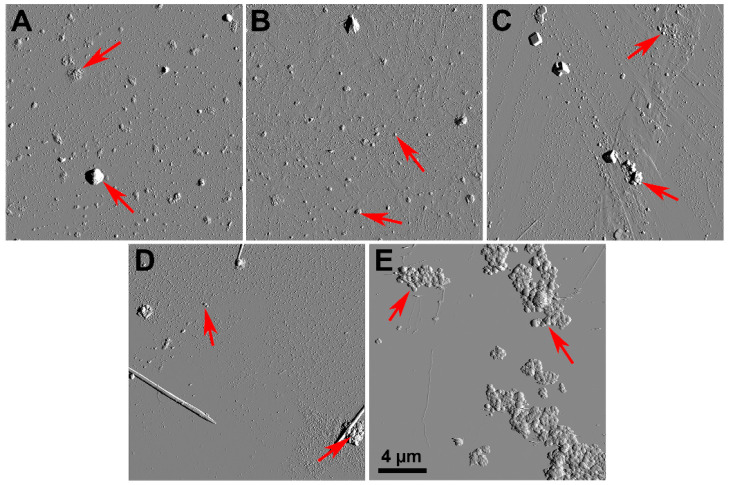
Atomic force microscopy images of fly ash samples from Rockdale (**A**); Dolet Hills (**B**); Rockport (**C**); Muskogee (**D**); Chelyabinsk (**E**).

The topography of the particles was studied using atomic force microscopy (AFM; Figure 1). Some of the samples disintegrated in water for a few minutes into smaller aggregates, and larger conglomerates were formed upon back drying during sample preparation for AFM (Figure 1A–D). The largest conglomerates were produced by Chelyabinsk particles (Figure 1E). Note that a spherical shape is typical for ash particles formed during coal or waste combustion [33,34,35]. The spherical topography is visible in the enlarged image (Figure S1). Spheres of 100–200 nm diameters were combined into agglomerates of few micrometer sizes (Figure 1A–E). Similar data on ash particle size distribution were reported elsewhere [25]. In addition to the topography, the adhesion of the studied particles was recorded. The surface of the analyzed fly ash particles was less adhesive (1–2 nN) than a clear glass surface (10–20 nN).

In general, the particles were spherical; however, certain inclusions of crystals of square and tubular morphologies were also detected. Using AFM operating in PeakForce Tapping mode, we demonstrated that in all specimens, the conglomerates’ sizes were less than 20 μm (during dispersion in water and upon drying). Ultrafine ash (<0.2 μm) was reported to be more toxic in vivo and in vitro [36], and size was a key determinant of toxicity of fly ash samples [37]. The smaller particle size determines its chemical and biological reactivity. Ultrafine fly ash samples (D ≤ 0.1 µm) have the highest impact on human health. They are able to penetrate the lungs or skin barriers and enter the bloodstream and the lymphatic system of humans, localizing inside organelles such as mitochondria and disrupting their function [38]. Most of the studied fly ash particles were larger than this critical size and could be safer.

**Figure 2:**
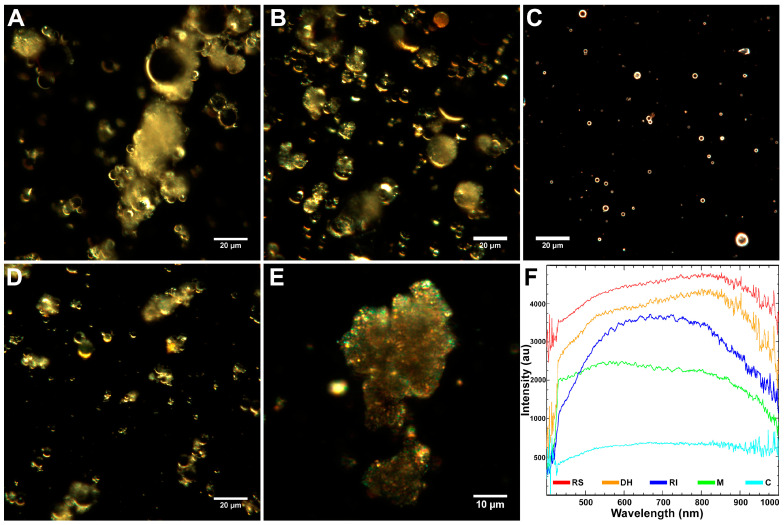
Fly ash samples visualized using dark-field microscopy: Rockdale—RS (**A**), Dolet Hills—DH (**B**), Rockport—RI (**C**), Muskogee—M (**D**), Chelyabinsk—C (**E**); reflected light spectra of fly-ash (**F**).

Additionally, the analysis of the morphology of fly ash samples was carried out using hyperspectral dark-field microscopy (Figure 2A–E). Figure 2 shows typical dark-field images of fly ash particles in distilled water. The particles were inhomogeneous, had irregular morphology, and Chelyabinsk particles formed large aggregates of ca 30 µm (Figure 2E). In addition, hyperspectral microscopy was used to characterize the fly ash samples spectrally. Spectral reflectance data were collected from fly ash samples in the visible and near-infrared range (400–1000 nm) with a spectral resolution of 2 nm. The spectral profiles were corrected for the light source’s spectral contribution using the internal correction algorithm of the ENVI software. 

The spectral signatures of fly ash particles are shown in Figure 2F: Rockdale particles had a broad peak in the 430–1000 nm region with a maximum at 800 nm, Dolet Hills particles had bimodal peaks at 500 and 800 nm, Rockport particles demonstrated narrow peaks at 550, 600, 650, 730, and 850 nm. Muskogee particles spectrum showed an extended maximum at 430 to 1000 nm with a characteristic peak at 600 nm. Chelyabinsk particles showed a broad maximum at 430–1000 nm. The spectral libraries collected for these particles were used for the subsequent hyperspectral detection of fly ash in HeLa cells.

### 2.2. Cytotoxicity

**Figure 3:**
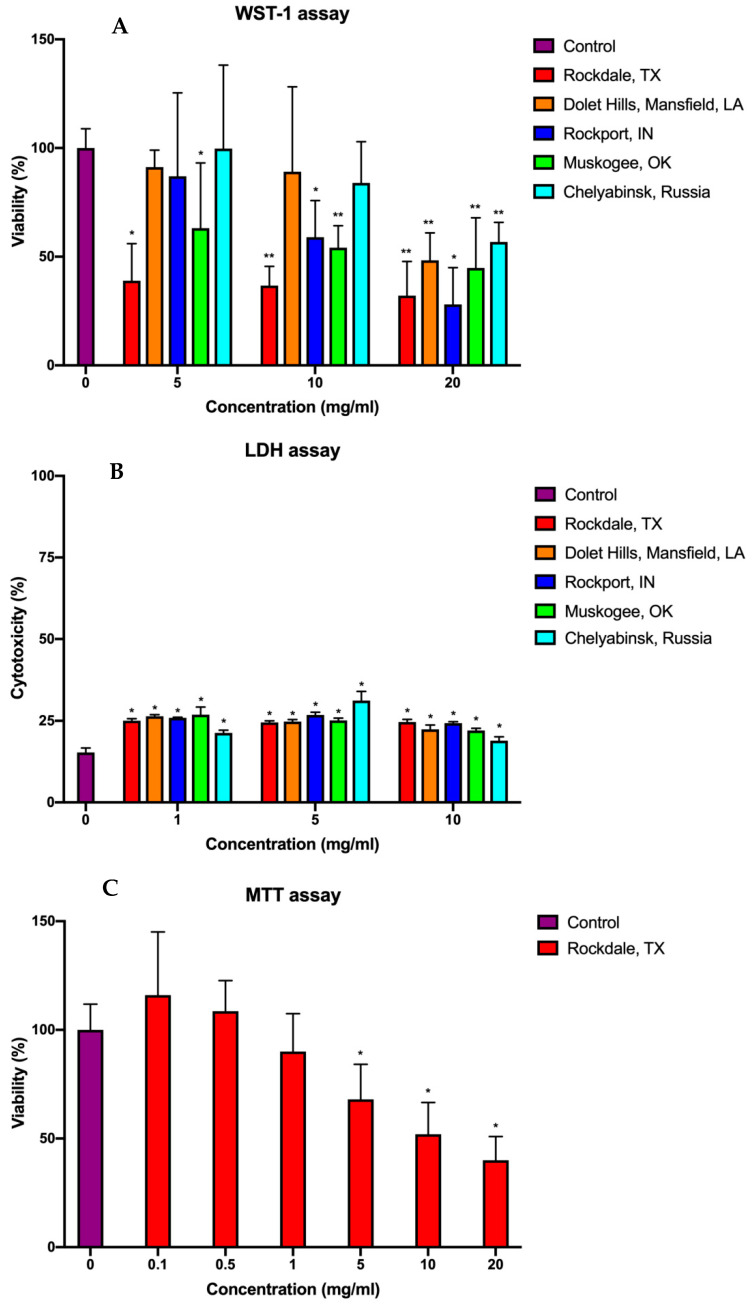
Relative cell viability and death of HeLa cells (human cervical cancer cells) after 24 h incubation with different concentrations of fly ash samples assessed by the WST-1 assay (**A**), LDH assay (**B**), and MTT assay (**C**). Results of WST-1 assay and MTT assay are expressed as a percentage of the untreated control group (mean ± standard deviation, * *p* < 0.05, ** *p* < 0.01). The LDH assay results are expressed as a relative percentage to the lysis buffer-treated group (mean ± standard deviation, * *p* < 0.05).

The fly ash samples’ cytotoxicity toward the human HeLa cell line was measured using the WST-1 assay. The viability was expressed as a percentage relative to that of cells in the untreated control. The concentration of fly-ash particles of different origins was varied from 5 to 20 mg/mL. Incubation of cells with ultra-high concentrations of particles for 24 h caused a dose-dependent decrease in viability in all studied groups (Figure 3A).

The most prominent cytotoxic effect at a concentration as low as 5 mg/mL was produced by the ash from Rockdale and Muskogee. After exposure to Rockdale and Muskogee particles at 5 mg/mL, the cell viability was 38 and 63%, respectively. When the concentration was increased to 20 mg/mL, the ash samples from Rockdale and Muskogee dose-dependently inhibited the viability to 32 and 45%, respectively. Meanwhile, the other samples at the concentration of 5 mg/mL showed an insignificant change of cell viability compared to control. The ash from Dolet Hills and Chelyabinsk, even at the concentration of 10 mg/mL, has no significant toxic effect on cancer cells’ viability. Expectedly, the same samples had the least cytotoxicity at the maximum concentration. The fly ash from Rockport was shown to be comparable with Muskogee sample level of cytotoxicity at 10 mg/mL. However, when the concentration was increased to 20 mg/mL, the cell viability was the lowest among all groups. Using WST-1 tetrazolium salt as an indicator of cell viability, we showed a dose-dependent cytotoxic effect of all studied coal fly ash samples. However, the findings suggest that both class C and class F fly ashes at the ultra-high concentrations could inhibit the metabolic function of the cells at a similar level. Notably, it was found that the particles forming the largest aggregates revealed by microscopy were the least cytotoxic over the whole range of concentrations. In the same studies of the toxicity of airborne particulate matter ranging from coarse (>2.5 μm) to ultrafine (<0.2 μm) at a range of concentrations 25–250 µg/mL, significantly higher toxicity of ultrafine particles was also revealed [36,37]. 

The analysis of lactate dehydrogenase (LDH) release after the cultivation of HeLa cells with coal fly ash particles for 24 h showed no dose-dependent response in a majority of samples (Figure 3B). The cytotoxicity level was increased from 15% in the control group to ≈25% in experimental group cultivated with the samples obtained from USA plants at the concentration of 1 mg/mL. The increase of concentration to 5 and 10 mg/mL did not influence the cytotoxicity, keeping it at the same level. On the other hand, the cytotoxicity of the coal fly ash from Chelyabinsk increased from 21 to 31% with the increase of the concentration of particles from 1 to 5 mg/mL. However, at the 10 mg/mL, the cytotoxicity level dropped to 19%. The other studies applied LDH assay on macrophage cell cultures treated with fly ash samples and demonstrated a similar plateau for ashes with the fine particle size at a range of concentrations up to 250 µg/mL [37,38]. Moreover, as previously reported by Kaw et al., the exposure of coal fly ash from different power plants to macrophages could produce the same level of enzyme release [39]. Although no dose-dependent effect on the cell viability was observed in LDH assay, the treatment with all fly ash samples resulted in significant cell membrane damage at all concentrations studied.

Additionally, the assessment of cell viability using the MTT (3-(4,5-dimethylthiazol-2-yl)-2,5-diphenyl tetrazolium bromide) assay was performed on the HeLa cell line exposed to the fly ash sample from Rockdale that produced the most acute effect at the 1 mg/mL group in WST-1 assay. The metabolic activity in the concentration range of 0.1–20 mg/mL shown in Figure 3C has clear dose-dependency with a significant reduction of viability started at 5 mg/mL. The highest concentration of Rockdale sample reduced the viability of the cells to 40%. The variance in cell viability results could be explained by the different mechanism of reduction of the tetrazolium salts: the WST-1 (water-soluble tetrazolium; 4-[3-(4-iodophenyl)-2-(4-nitrophenyl)-2H-5-tetrazolio]-1,3-benzene disulfonate) reagent does not penetrate the cell membrane, being reduced extracellularly, while the MTT salt is able to pass through the membrane and is reduced by mitochondrial enzymes [40]. Thus, the fly ash contained in media and the surrounding cells may interfere with the reduction of WST-1, decreasing cell viability compared to MTT assay results.

The pH of the medium containing fly ash particles can also affect cell viability. The pH of the aqueous and culture medium extracts of fly ash particles was measured (Table S2). These values are in good agreement with the alkaline pH values of fly ash leachates reported previously [41,42]. However, no correlation between the pH value and cytotoxic effects of fly ash species was observed, suggesting that low added amount of ash did not change essentially the pH of the cell environments.

### 2.3. Genotoxicity

**Figure 4:**
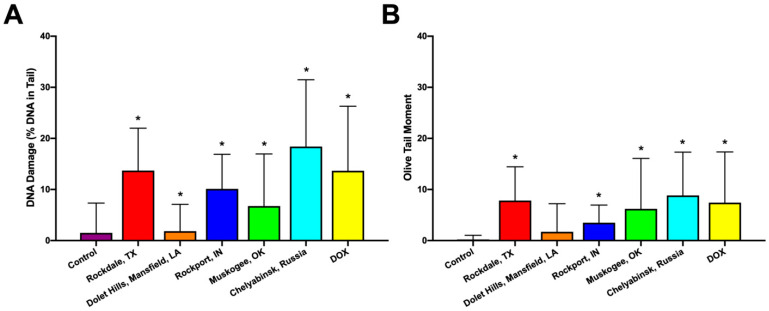
Genotoxicity assay indicating the damage of HeLa cells DNA after 24 h of exposure to fly ash particles (5 mg/mL): tail lengths of DNA comets (**A**); Olive tail moments of DNA comets (**B**). * *p* < 0.05 when compared with the control group.

Based on the cytotoxicity results, the concentration of fly ash particles equal to 5 mg/mL was chosen for further genotoxicity analysis by single-cell electrophoresis (DNA-comet assay). Single- and double-stranded DNA breaks resulting from cell exposure to fly ash samples lead to the appearance of broken DNA molecules migrating at an increased speed under the electric field and the formation of comet-like structures (Figure 4A,B). The spatial parameters of DNA-comets manifest the extent of nuclear DNA destruction into low-molecular-weight fragments by the genotoxic compound. The presence of a long tail in such comets correlates with the effect of the test substance on the DNA molecule’s integrity and indicates the presence of a genotoxic effect (Figure 4A,B).

As shown in Figure 4, a statistically significant increase in the percentage of DNA in tails was observed in all fly ash samples (Figure 4A). Interestingly, that the Chelyabinsk sample had the greatest negative impact on the DNA structure. It was noted that genotoxicity of fly ash may be caused by generation of reactive oxygen species (ROS) due to presence of trace elements, Fe, SiO_2_, and Al_2_O_3_ in comp osition [43]. Our data correlate well with earlier data reporting that the fly ash particles and leachates induced DNA damage in human peripheral blood mononuclear cells [44] and whole blood cells and lymphocytes [45].

### 2.4. Flow Cytometry

Since the attached HeLa cells come into contact with a medium containing fly ash with only one of their surfaces and to additionally investigate the effects of fly ash on different cell types, fly ash toxicity at various concentrations was evaluated on human Jurkat T suspension cells using flow cytometry (Table 3). Adaptive immunity is known to be mainly mediated by lymphocytes, and there are few studies evaluating fly ash toxicity to lymphocytes.

The negative effect of fly ash for suspension cell culture was dose-dependent, similarly to other studies [35], with Chelyabinsk fly ash demonstrating the highest cytotoxic effect. In this study, cancer human cells were used as in vitro models characterized by uncontrolled and rapid growth, requiring a large number of nutrients from the environment, which increases their sensitivity to fly ash components. Annexin is known to detect phosphatidylserine translocation to the outer layer of the cell membrane during apoptosis, while PI marks late apoptotic and necrotic cells that have lost membrane integrity [46]. According to the flow cytometry data, the toxic effects of fly ash were manifested by necrosis and later apoptosis (Figure S1). An increased cell death rate through necrosis and apoptosis was earlier observed in V79 (Chinese hamster lung fibroblasts) cell line treated with high concentrations of coal fly ash [43]. 

### 2.5. Fly Ash Biodistribution

**Figure 5:**
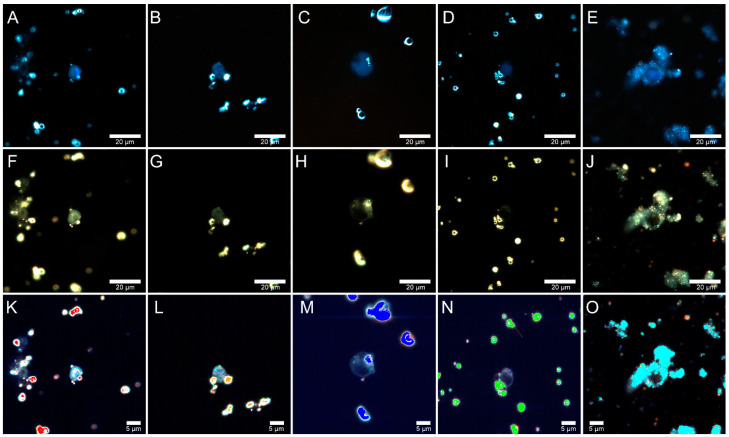
Merged dark-field and fluorescence (upper row) images of Jurkat cells, dark-field images (middle row), and corresponding hyperspectral images merged with maps (bottom row) demonstrating distribution of fly ash particles in living Jurkat cells incubated with 1 mg/mL concentrations of particles from Rockdale (**A**,**F**,**K**); Dolet Hills (**B**,**G**,**L**); Rockport (**C**,**H**,**M**); Muskogee (**D**,**I**,**N**); and Chelyabinsk (**E**,**J**,**O**) fly ash particles.

Additionally, hyperspectral images of Jurkat cells incubated for 24 h with fly ash particles at concentrations of 1 and 5 mg/mL were obtained. Jurkat cells were visualized using a CytoViva dual fluorescence module (CytoViva, Auburn, AL, USA). An excitation light source, an X-cite 120Q wide-field fluorescence lamp (Excelitas Technologies, Mississauga, Canada), and a CytoViva^®^ dual-mode fluorescence module with a triple pass filter were used to visualize the fluorescent staining of cell nuclei, and the exposure time was 10 s. The resulting dark-field images were combined with transmission fluorescence images using the free GIMP 2.10.18 software. The images of fly ash particles in Jurkat cells after 24 h co-incubation at a concentration of 1 mg/mL are shown in Figure 5. Fluorescence (Figure 5A–D) and dark-field (Figure 5G–J) images showed those fly ash particles from Rockdale, Dolet Hills, and Muskogee did not penetrate the cells, but they were located as single particles on the cell surface, which was perhaps due to the large particle sizes. However, Rockport and Chelyabinsk fly ash particles penetrated into cells and were found in the cell cytoplasm. The particles were small enough to cross the membrane and to form small aggregates in the cytoplasm. Next, the hyperspectral characterization of fly ash in Jurkat cells was carried out using an internal spectral mapping algorithm (Figure 5K–O). Hyperspectral mapping of the Dolet Hills, Rockdale, and Muskogee fly ash particles in Jurkat cells confirmed that the particles were present on the cell surface (Figure 5L–N). However, one can see that Rockport and Chelyabinsk particles corresponded to more pixels within cells (red and cyan pixels in hyperspectral images, respectively), suggesting better cell penetration by these particles.

**Figure 6:**
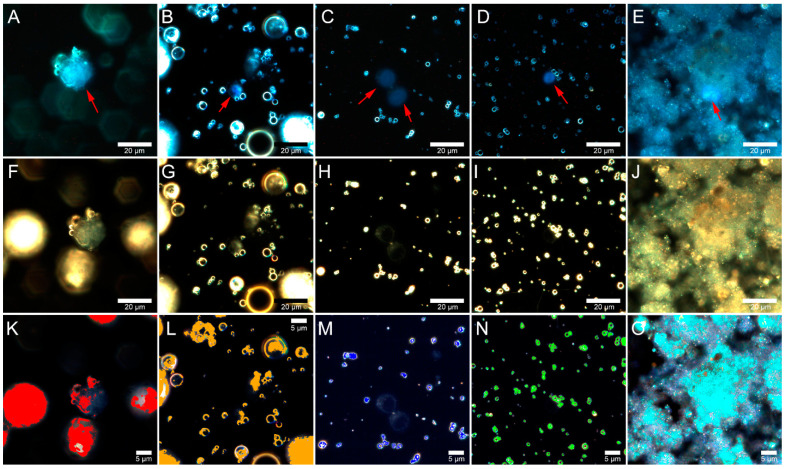
Merged dark-field and fluorescence (upper row) images of Jurkat cells, dark-field images (middle row), and corresponding hyperspectral images merged with maps (bottom row) demonstrating the distribution of fly ash particles in living Jurkat cells incubated with 5 mg/mL concentrations of particles from Rockdale (**A**,**F**,**K**); Dolet Hills (**B**,**G**,**L**); Rockport (**C**,**H**,**M**); Muskogee (**D**,**I**,**N**); and Chelyabinsk (**E**,**J**,**O**) fly ash particles. Red arrows point to Jurkat cells.

Next, Jurkat cells were examined after incubation with fly ash particles at a 5 mg/mL concentration for 24 h (Figure 6A–O). For Rockdale and Chelyabinsk particles, an increase in concentration resulted in increased uptake and intracellular distribution after 24 h of incubation. Hyperspectral mapping confirmed that Rockdale particles were bound to cells, as indicated by red pixels in hyperspectral images. Hyperspectral mapping of Dolet Hills fly ash showed the presence of particles in the cell cytoplasm (Figure 6L). In contrast, the fluorescence and dark-field images showed only single large particles on the cell surface (Figure 6B,G).

Fly ash particles from Dolet Hills (Figure 6B–L), Rockport (Figure 6C–M) and Muskogee (Figure 6D–N) were not visible in the cell cytoplasm, while fly ash from Rockdale (Figure 6A–K) and Chelyabinsk (Figure 6E–O) penetrated the cells; single fly ash particles or small agglomerates were present in the cytoplasm. Thus, cell penetration can probably explain the high toxicity of the samples shown by flow cytometry. Using dark-field and hyperspectral microscopy, the distribution and formation of aggregates of air pollutants in lungs [47,48,49], human embryonic stem cells [50], cultures of macrophages, and bronchial epithelial cells [51] were studied. We were the first to study the distribution and uptake of fly ash in Jurkat cells, and with the help of hyperspectral microscopy, the fly ash particles were found either on the surface or inside the cells. 

**Figure 7:**
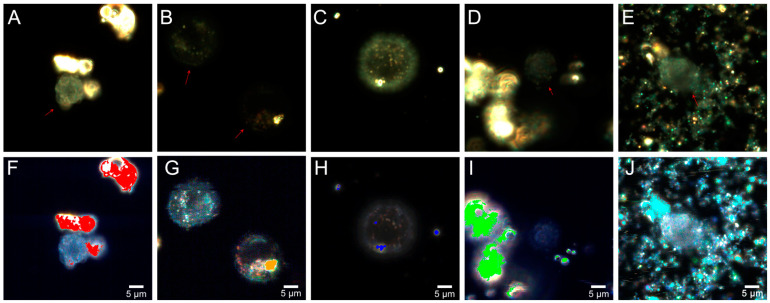
Dark-field images (upper row) and corresponding hyperspectral images merged with maps (bottom row) demonstrating distribution of fly ash particles in living HeLa cells after 1 h of co-incubation: Rockdale (**A**,**F**); Dolet Hills (**B**,**G**); Rockport (**C**,**H**); Muskogee (**D**,**I**); and Chelyabinsk (**E**,**J**).

The distribution of fly ash particles in HeLa cells was also assessed using hyperspectral microscopy during the first hours of cell interaction with fly ash samples. Typical dark-field images in Figure 7 illustrate the fly ash in HeLa cells after co-incubation at 5 μg/mL for 1 h. The ash samples scattered light with high efficiency and appeared as bright white structures in dark-field images, while cells (shown with red arrows) were significantly dimmer. In HeLa cells exposed to Muskogee particles for 1 h (Figure 7D–I), the microparticles did not penetrate the cells and were predominantly located outside. On the contrary, particles from Rockdale (Figure 7A–F), Dolet Hills (Figure 7B–G), Rockport (Figure 7C–H) and Chelyabinsk (Figure 7E–J) were found inside the cytoplasm. Large accumulations of fly ash particles in HeLa cells were observed for Rockdale particles (Figure 6), which is consistent with our data on the cyto- and genotoxicity. Then, a hyperspectral mapping technique was applied to detect fly ash in HeLa cells. The few Rockdale particles were found inside the cells, as indicated by red pixels in the hyperspectral image. When cells were treated with Chelyabinsk particles, cyan spots were imaged inside the cells, indicating that some of the fly ash particles were internalized. Fewer Dolet Hills and Rockport particles, as represented by orange and blue pixels, were found in the cell compared to Rockdale and Chelyabinsk particles. No internalized Muskogee particles were seen within the cells.

**Figure 8:**
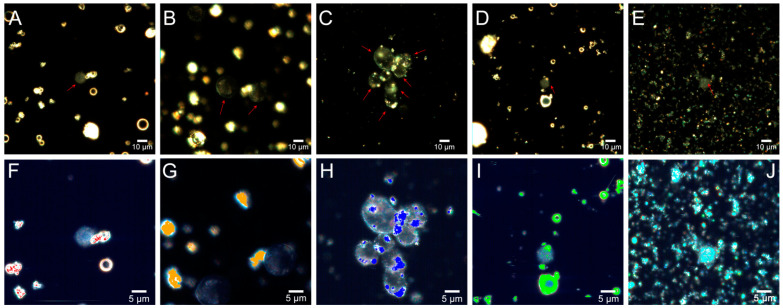
Dark-field images (upper row) and corresponding hyperspectral images merged with maps (bottom row) demonstrating distribution of fly ash particles in living HeLa cells after 3 h of co-incubation: Rockdale (**A**,**F**); Dolet Hills (**B**,**G**); Rockport (**C**,**H**); Muskogee (**D**,**I**); Chelyabinsk (**E**,**J**).

Figure 8 shows dark-field images of a HeLa cell suspension culture after incubation with fly ash particles at the concentration of 5 mg/mL for 3 h. Prolonged exposure to fly ash microparticles did not lead to changes in the cell morphology. Large accumulations of the particles were visible as bright spots in the cytoplasm of HeLa cells and were found in Rockport (Figure 8C–H), Muskogee (Figure 8D–I), and Chelyabinsk samples (Figure 8E–J). At the same time, Rockdale and Dolet Hills particles were discernible only on the cell surfaces. Muskogee particles were detected in the cell cytoplasm after 3 h of exposure (Figure 8D,I).

The penetration of fly ash particles most probably occurs during the first hours of their interaction with cells. Figure 8 (bottom row) shows a hyperspectral image with an overlaid spectral angle map. Spectral mapping results also confirmed that aggregates were formed in HeLa cells treated with fly ash. When dispersed in water, ash particles are smaller in size than the cell and its organelles, which allows them to penetrate the cell constituents and disrupt their functions, causing tissue inflammation and shifting the redox balance toward oxidation, which may result in cell death [52].

**Figure 9:**
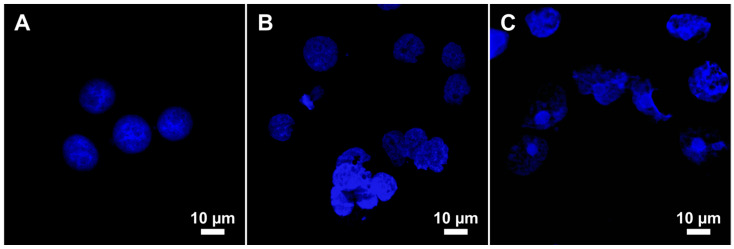
Confocal laser scanning microscopy images demonstrating the nuclei of HeLa cells without treatment (**A**) and after 24 h treatment with 5 mg/mL of fly-ash particles from Rockdale (**B**) and Chelyabinsk (**C**). The nuclei were stained with 4′,6-diamidino-2-phenylindole (DAPI).

Furthermore, the effect of the fly ash entering HeLa cells on nuclear morphology was examined using confocal microscopy (Figure 9). Analysis of the images showed that the introduction of Rockdale and Chelyabinsk fly ashes resulted in changes in the nuclei sizes and disruption of their normal morphology (Figure 9B,C), which coincides with the data of the cell proliferative activity and the effect on DNA damage. Control HeLa cells contained regularly shaped nuclei with smooth borders (Figure 9A). In cells incubated with fly ash, a number of nuclei having aberrant morphology were about 55–60% with the appearance of irregular, ruffled, and folded structures (Figure 9B,C).

### 2.6. Interaction Force between Mammalian Cell and Fly Ashes

**Figure 10:**
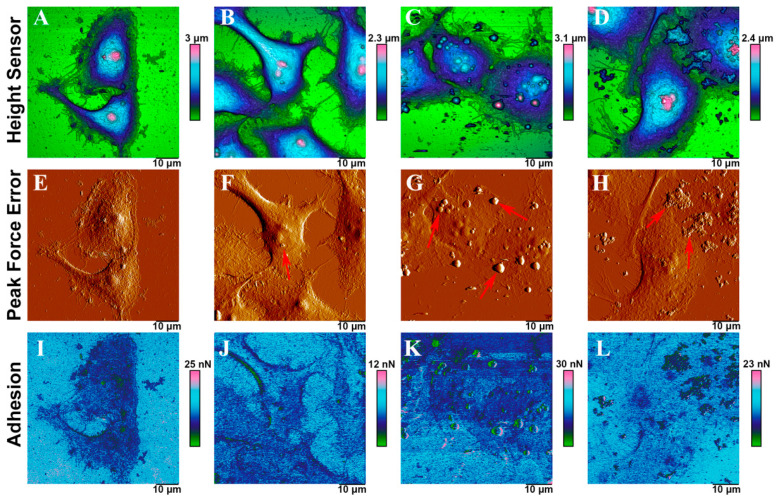
Atomic force microscopy (AFM) images of HeLa cells after 24 h incubation with 5 mg/mL of fly ash particles obtained in Height Sensor (upper row), Peak Force Error (middle row), and Adhesion (bottom row) channels: intact HeLa cells (**A**,**E**,**I**); HeLa cells treated with Rockdale (**B**,**F**,**J**),Rockport (**C**,**G**,**K**),and Chelyabinsk (**D**,**H**,**L**) ash particles. Red arrows indicate fly ash particles.

The localization of the ash microparticles on the surface of HeLa cells was investigated using AFM (Figure 10A–L). Atomic force microscopy does not allow visualizing particles inside human cells but makes it possible to observe them on the cell surface. In the Height Sensor channel, ash particles were visible on the surface of HeLa cells (Figure 10). We visualized some of the particles on the cell membrane; as shown using hyperspectral microscopy, these fly ash species were also located inside the cells. The particles were better seen in the Peak Force Error channel (marked with red arrows). In non-specific adhesion maps, these particles produced darker areas compared to the cell itself, since they were less adhesive. The ash microparticles were observed in the same positions in the correlated images, which indicated the particles’ attachment to the surface membrane of HeLa cells.

Cells incubated with fly ash particles did not differ in morphology from control cells (Figure 10A–I), regardless of the particle localization. However, the morphology of HeLa treated with Chelyabinsk fly ash could not be determined, since these particles were tightly adsorbed on the cell surface, making the force contouring difficult (Figure S2). Since dark-field microscopy visualized the interaction of the cell membrane with fly ash particles, the changes in the properties of the cell surface after 24 h of co-incubation with fly ash samples were further evaluated using atomic force microscopy. The cells were fixed and washed with phosphate-buffered saline and distilled water 3 times each, making it possible to evaluate the adhesion of particles tightly adherent to cells (Table 4).

We found that the fly ash from Rockport and Muskogee was more associated with cell membranes. Additionally, both of these species were also found in the cytoplasm of cells, as shown by hyperspectral microscopy. It is necessary to consider the size, morphology, and elemental composition and carry out a comprehensive assessment of the toxicity of fly ash samples at the cellular level [53]. For the first time, we used hyperspectral microscopy to assess the penetration of fly ash particles into human cells. We found that after 3 h of co-incubation, not all fly ash types were able to penetrate the cell membrane. Taken together, all studied fly ash species were, to a certain extent, cytotoxic to HeLa and Jurkat cells, although the degree of toxicity varied depending on the cell type and the test used. The Chelyabinsk fly ash sample was more toxic than other fly ash samples when studied in Jurkat cells, while this effect was not observed in HeLa cells. This discrepancy can be explained by variable susceptibility of different cell types to the same impacts, which is often observed in cell culture studies [54,55]. According to previous studies, the size of ash particles is crucial for their toxic effect on the body, and smaller particles are more harmful to human health [56,57]. In this study, the smallest particles (those obtained from Chelyabinsk and Rockport) demonstrated the highest cell penetration but different cytotoxicity in cultured Jurkat cells. Thus, the particle size was probably not the only determinant of the toxicity of fly ash samples studied. Additionally, no direct correlation could be traced between the chemical composition and toxicity of a given fly ash sample, suggesting that toxic effects resulted from the complex combination of different fly ash components as it was previously observed in other studies [58].

## 3. Materials and Methods

### 3.1. Materials

Fly ash samples in powder form were collected from different power plants in the United States: Rockdale, TX; Dolet Hills, Mansfield, LA; Rockport, IN; Muskogee, OK [59]. Additionally, a sample of fly ash was obtained from Chelyabinsk Electro-Metallurgical Works OJSC, Russian Federation. L-glutamine, penicillin, streptomycin, and MTT reagent were purchased from Sigma-Aldrich (Darmstadt, Germany). A Pierce LDH cytotoxicity assay kit was obtained from Thermo Scientific. The collected powders of samples were dispersed in Millipore water (specific resistivity 18 MΩcm at 25 °C), and the obtained solutions were placed in the ultrasonic bath for 15 min before experiments. The pH of aqueous and biological solutions of fly ash particles was measured using Seven Compact pH/Ion meter S220 (Mettler Toledo, Greifensee, Switzerland).

### 3.2. Cell Culture

The human cervical carcinoma (HeLa) cell line was purchased from American Type Culture Collection (ATCC; Manassas, VA, USA). Human T lymphocyte cells (Jurkat cells) were obtained from the Russian Cell Culture Collection (Saint-Petersburg, Russian Federation). Cells were cultured in Dulbecco’s Modified Eagle’s Medium (DMEM; Gibco, Gaithersburg, MD, USA) supplemented with 10% fetal bovine serum (Invitrogen, Carlsbad, CA, USA), 2 mM L-glutamine, and 1% penicillin−streptomycin solution at 37 °C in a humidified atmosphere with 5% CO_2_. HeLa cells were grown as monolayers in a 25-cm^2^ flask. Once the cells reached 80% confluence, they were passaged using the trypsin with ethylenediaminetetraacetic acid (trypsin−EDTA) solution and then incubated for 2−3 days before experiments [60]. Tali Image-Based cytometer (Invitrogen, Carlsbad, CA, USA) was used for cell counting.

### 3.3. WST-1 Assay

The effects of coal fly ash samples on cell viability were determined using a colorimetric assay, based on the reduction of the tetrazolium salt WST-1 (4-[3-(4-Iodophenyl)-2-(4-nitro-phenyl)-2H-5-tetrazolio]-1,3-benzene sulfonate) into formazan by cellular enzymes. WST-1 assay was performed following the manufacturer’s protocol (Roche, Basel, Switzerland). Briefly, HeLa cells were plated onto a 96-well plate at a density of 1 × 10^4^ cells per well containing 100 μL of culture medium. The next day, the culture medium from each well was replaced with fresh medium containing coal fly ash samples at different concentrations (5, 10, and 20 mg/mL). The cells cultivated without fly ash particles were considered as the untreated control. After 24 h of incubation at 37 °C, 10 μL of WST-1 reagent was added to the cells in each well. Following 4 h incubation at 37 °C, the optical density values were measured at 440 and 690 nm using a Multiskan™ FC Microplate Photometer (Thermo Fisher Scientific, Waltham, MA, USA).

### 3.4. LDH Assay

Lactate dehydrogenase (LDH) release after the incubation of HeLa cells with different concentrations of samples (1, 5, 10 mg/mL) was measured to check if the coal fly ash influences cell membrane integrity. The assay was conducted according to the manufacturer’s instructions (Thermo Fisher Scientific, Waltham, MA, USA). The cells were seeded onto a 96-well plate at density 1 × 10^4^ cells per well containing 100 µL of culture medium. The next day, the culture medium was replaced with medium containing different coal fly ash concentrations, and the cells were cultured for 24 h. The cells incubated without fly ash particles were considered as the untreated control. Then, 50 µL of the medium from wells was transferred to a new plate, which was followed by the subsequent addition of 50 µL of LDH reaction mixture to each well. After incubation for 30 min at RT, the 50 µL the stop solution was added to all sample wells. The optical density values were measured at 485 nm using FLUOstar Omega (BMG Labtech, Ortenberg, Germany). The cytotoxicity was expressed as percent relative to absorbance values of lysis buffer-treated cells. 

### 3.5. MTT Assay

Additionally, the viability of cells incubated with coal fly ash from Rockdale, TX was measured using the MTT assay. HeLa cells were plated onto a 96-well plate at a density of 2 × 10^3^ cells per well with 200 μL of culture medium. After 24 h of incubation, the medium in each well was replaced with fresh medium containing coal fly ash samples at examined concentrations (0.1, 0.5, 1, 5, 10, and 20 mg/mL). The cells cultivated without fly ash particles were considered as the untreated control. The next day, the cells were washed with phosphate-buffered saline (PBS), and 200 μL of fresh medium with 20 μL of MTT reagent (5 mg/mL solution; Sigma-Aldrich, Darmstadt, Germany) was added to each well [61]. After 4 h of incubation, the culture medium was replaced with 200 μL of DMSO. The optical density values were measured at 540 nm using Multiskan™ FC Microplate Photometer.

### 3.6. Comet Assay

DNA damage was evaluated by the alkaline comet assay. HeLa cells at a density of 10^5^ cells per well were seeded onto 6-well plate. After 24 h of incubation at 37 °C, the different coal fly ash samples at a concentration of 5 mg/mL were added to the cells. The doxorubicin (2 mg/mL) was used as a positive control. The cells cultivated without fly ash particles were considered as a negative control. The next day, the cells were collected from plates and mixed with low-melting-point agarose (1.5%). The cells in 120 μL (3 × 10^4^ cells) agarose were smeared on glass slides precoated with normal melting point agarose (1%). After solidification, the cells were lysed with Triton lysis buffer (10 mM Tris, 2.5 M NaCl, 100 mM EDTA, 1% Triton X-100; pH 10) for 45 min at 4 °C in dark conditions. After lysis, the slides were placed in the alkaline solution (1 mM EDTA, 300 mM NaOH; pH 13) to allow DNA unwinding and subjected to electrophoresis (20 V, 300 mA; 30 min) in the same solution. The slides were soaked in 70% ethanol for 5 min for DNA fixation. Finally, the slides were stained using ethidium bromide for 15 min and analyzed using confocal microscopy (LSM 780; Carl Zeiss, Oberkochen, Germany) and CometScore 2.0 software (Tritek Corp., Sumerduck, VA, USA). The results were expressed as the percentage of DNA in the tail averaged from 40 randomly selected cells per slide (2 slides per sample).

### 3.7. Flow Cytometry

Jurkat cells were plated in 6-well plates (1 × 10^5^ cells per well) and incubated for 24 h. The fly ash samples were added to the cells at different concentrations, and the cells were further incubated for 24 h. The cells were collected, washed twice with PBS, stained with Annexin V-FITC (fluorescein isothiocyanate; apoptosis, green) and PI (propidium iodide) [62], and analyzed on FACS Aria III flow cytometer (BD Biosciences, San Jose, CA, USA). Cells were analyzed for Annexin V (An) binding and propidium iodide (PI) incorporation to distinguish between apoptotic (An+/PI−) and late apoptotic/necrotic (An+/PI+ and An−/PI+) cells.

### 3.8. Preparation of Samples for Microscopic Analysis

Jurkat cells were harvested by centrifugation at 800 RPM for 5 min and seeded onto 48-well plates at a density of 2 × 10^5^ per well containing 400 µL of medium with coal fly ash samples (at the concentration 1 or 5 mg/mL). After 24 h of incubation under continuous shaking at 50 RPM, the cells were fixed in a 4% paraformaldehyde solution that was added to each well. Then, the suspension was transferred to a 2 mL centrifuge tubes, and the cells were washed three times with PBS and cell nuclei were stained with 4′,6-diamidino-2-phenylindole (DAPI) according to the standard protocol. Then, the cells were washed with PBS and two times with diH_2_O. A drop of the cell suspension was pipetted onto a coverslip, which was placed onto the slide with mounting medium on its surface. In addition, the biodistribution of coal fly ash in the HeLa cell line was analyzed microscopically within the cells suspended in growth medium and exposed to the particles at the concentration of 5 mg/mL for 1 or 3 h. A drop of the cell suspension was placed on a microscope slide, which was then carefully sealed with a coverslip and nail polish. Hyperspectral imaging was carried out immediately after the samples were prepared. Alternatively, HeLa cells were exposed to coal fly ash particles while still attached to the substrate. Briefly, cells at a density of 5 × 10^4^ per well were seeded onto 6-well plates with round coverslips on the bottom of each well. The following day, the growth medium was replaced with fresh growth medium containing coal fly ash particles at the concentration 1, 5, or 10 mg/mL. After 24 h of incubation, the cells were washed with PBS and fixed with 3.7% paraformaldehyde for 15 min. The cell nuclei were stained with DAPI according to the standard protocol. Then, the coverslip was placed on a microscope slide with a drop of mounting medium on its surface. Then, the morphology of HeLa cells was analyzed by dark-field and confocal microscopy. 

### 3.9. Dark-Field Microscopy and Hyperspectral Imaging

The distribution of fly ash particles in Jurkat and HeLa cell lines was visualized with a CytoViva Enhanced Darkfield Hyperspectral Imaging System (CytoViva Inc., Auburn, AL, USA). Dark-field images and spectra of reflected light were obtained using Olympus BX51 upright microscope (Olympus, Tokyo, Japan), equipped with a CytoViva^®^ enhanced dark-field condenser (CytoViva, Auburn, AL, USA), halogen light source (Fiber-Lite DC-950, 150W; Dolan Jenner Industries Inc., Boxborough, MA, USA), and ProScan III controller (JH Technologies, Fremont, CA, USA). Dark-field images were obtained using Exponent 7 software (Stable Microsystems, Godalming, UK). Hyperspectral images were collected at the visible and near-infrared (VNIR) range (400–1000 nm) with a step of 2 nm using ImSpector V10E spectrograph (Specim, Oulu, Finland) and a charge-coupled device (CCD) camera (PCO AG, Kelheim, Germany). Spectral libraries were built using ENVI 4.8 software (Harris Geospatial Solutions, Broomfield, CO, USA). The pure samples of coal fly ash particles (*n* = 20) were used to collect reference spectra. Then, hyperspectral images of the Jurkat and HeLa cell lines treated with coal fly ash particles were obtained for determining the distribution of particles at different time points. The internal spectral mapping algorithm was used to confirm spectral coincidence between image pixels in hyperspectral datacube of cells and spectral libraries of pure particles [63,64,65,66]. The spectral angle coefficient for HeLa cells treated with particles for 1 h was 0.6 (Rockdale, Dolet Hills), 0.5 (Rockport), and 0.2 (Muskogee, Chelyabinsk) rad. The spectral angle coefficient for HeLa cells treated with particles for 3 h was 0.16 (Rockdale), 0.3 (Dolet Hills), 0.7 (Rockport), 0.43 (Muskogee), and 0.7 (Chelyabinsk) rad. The fluorescent images of the cells were collected using an X-cite 120Q wide-field fluorescence lamp (Excelitas Technologies, Mississauga, Canada) and a CytoViva® dual-mode fluorescence module with a triple pass filter.

### 3.10. Atomic Force Microscopy

AFM analysis was performed with the atomic force microscope Dimension Icon (Bruker, Billerica, MA, USA) [67]. The samples of pure particles were prepared by the drop-cast method of particle suspension on ultrapure glass slides with subsequent drying at RT. The cells treated with coal fly ash for AFM analysis were prepared as follows. HeLa cells at a density of 10^5^ cells per well were seeded onto a 6-well plate containing round glass coverslips in each well. The following day, the culture medium from each well was replaced with 1 mL of fresh medium containing different coal fly ash samples at a 5 mg/mL concentration. After 24 h of incubation, the cells were fixed with 4% paraformaldehyde, washed with ultrapure water, and air-dried. Images were collected in the PeakForce Tapping mode. ScanAsys-air probes (nominal length 115 μm, tip radius 2 nm, nominal spring constant 0.4 N m^−1^; Bruker) were applied to analyze the samples in air. The raw AFM data were processed using the Nanoscope Analysis v.1.7. software (Bruker). For each sample, the adhesion forces between the cell surface and the cantilever needle were determined. Quantitative calculation of adhesion was carried out in the Nanoscope Analysis v 1.7 software (Bruker) in the adhesion channel. The adhesion images were not pre-edited to obtain reliable results. The surface adhesion of cells with particles was calculated from 15 square areas of the same size (20 µm × 20 µm) for each sample.

### 3.11. Fly Ash Analysis Techniques

The qualitative elemental composition of the fly ash samples was determined by energy-dispersive spectroscopy using an Oxford Instruments X-Max™ 80T silicon-drift detector. An Auriga Crossbeam (Carl Zeiss AG, Oberkochen, Germany) instrument (SEM) was used for fly ash analysis. The elemental composition of fly ash was identified also using a Bruker D8 X-ray diffractometer (Bruker, Karlsruhe, Germany). For pH measurements, 5 mg of fly ash was dispersed in a 1 mL distilled water or culture medium. Then, the mixture was sonicated for 30 s, and the pH of dispersions was measured. 

### 3.12. Statistics

Statistical analysis was conducted using GraphPad Prism v 8.4.3 (GraphPad Software, Inc., San Diego, CA, USA). The differences between the groups of cells exposed to different fly ash samples and the control group were determined using the Mann Whitney U test with statistical significance at *p* < 0.05.

## 4. Conclusions

An expansion of the industrial coal combustion, especially in developing countries, produces a mass of fly ash, which is mostly filtered and stored but partially leaks to the atmosphere. Although stricter regulations regarding the containment and storage of coal fly ash have been placed in some countries, such as the USA, both stored and airborne fly ash microparticles may pollute water and air, resulting in the need for research into its effects on human cells, especially since fly ash has been associated with the incidence of various types of cancers. Fly ash samples of five different origins were characterized with appropriate methods, and their in vitro cytotoxicity was examined. The toxicity of airborne fly ash particles inversely correlated with their size. The smallest particle size of 0.4–0.6 µm in water was observed for fly ash from Russia (Chelyabinsk), which turned out to be the most toxic to Jurkat cells and the most genotoxic to HeLa cells. However, no direct correlation could be traced between the size or chemical composition and toxicity of a given fly ash sample, suggesting that toxic effects resulted from the complex combination of different fly ash properties. At 5–10 mg/mL concentration of ash in water, the cell survival rate was above 55% for all studied fly ash from US power stations, indicating medium toxicity for Jurkat cells. However, the observed cytotoxic effects of fly ash depended on the cell culture and viability test used. Note that the concentration of 5 mg/mL (corresponds to 5000 g/m^3^) was used for all studied samples, which significantly exceeds the content of fly ash in the air. Even very polluted air does not have more than 1 mg/m^3^ fly ash microparticles [4,5]. Of course, water may accumulate a lot of hydrophilic (ζ-potential ca −25 mV) fly ash microparticles for a long time. For the first time, spectral libraries of fly ash particles sampled from different industrial plants were created. Further detailed studies should be carried out at the organ level, including studying the effects of solid air particles on the lungs, liver, brain, and other organs and systems of mammals.

## Figures and Tables

**Table 1:** ζ-potentials and hydrodynamic characteristics of fly ash samples.

Sample Origin	Size, µm	ζ-Potential, mV
Rockdale, TX	2.7 ± 0.1	−28.1 ± 1.2
Dolet Hills, LA	2.0 ± 0.3	−12.3 ± 1.5
Rockport, IN	1.2 ± 0.3	−28.9 ± 2.1
Muskogee, OK	2.0 ± 0.1	−30.0 ± 2.9
Chelyabinsk, Russia	0.4 ± 0.2	−35.2 ± 0.6

**Table 2:** Elemental composition of fly ash particles in weight percent measured by energy-dispersive spectroscopy (EDS).

Elements	Rockdale	Dolet Hills	Rockport	Muskogee	Chelyabinsk
O	46.94	47.03	42.59	42.97	51.27
Si	25.79	26.19	14.89	17.37	43.06
Al	13.10	12.46	13.36	11.96	0.56
Na	0.22	1.39	5.52	0.92	0.63
Mg	0.90	1.58	1.76	4.07	1.00
S	0.43	0.50	2.21	0.59	-
K	1.19	0.76	0.55	0.36	1.34
Ca	6.20	5.31	8.13	17.17	0.28
Ti	1.04	0.40	1.22	1.14	-
Fe	3.83	3.71	2.32	3.26	0.93

**Table 3:** Viability of Jurkat T cells after 24 h incubation with fly ash samples at different concentrations evaluated with flow cytometry. The cells were stained with fluorescein isothiocyanate-coupled annexin V (An) and propidium iodide (PI).

Sample Origin	Ash Concentration, mg/mL	Live, %	An+\PI+, %	An+, %	PI+, %
Control	-	97.8 ± 0.4	0	2.2 ± 0.1	0
Rockdale	1	69.7 ± 0.8	21.1 ± 1.1	1.3 ± 0.1	8.0 ± 0.3
	5	65.2 ± 2,1	19.6 ± 0.7	1.9 ± 0.1	13.5 ± 0.4
	10	61.9 ± 0.4	17.9 ± 0.9	2.0 ± 0.2	18.3 ± 1.5
Dolet Hills	1	68.4 ± 0.6	22.2 ± 1.9	1.3 ± 4.8	8.2 ± 2.7
	5	66.7 ± 1.6	19.0 ± 1.0	1.5 ± 5.4	12.9 ± 1.2
	10	63.0 ± 1.2	17.7 ± 0.2	1.8 ± 5.0	17.5 ± 2.3
Rockport	1	71.6 ± 3.1	19.5 ± 0.4	1.6 ± 6.2	7.3 ± 1.4
	5	67.6 ± 2.1	19.7 ± 0.9	1.6 ± 5.8	11.2 ± 1.4
	10	63.8 ± 0.5	19.5 ± 0.4	1.8 ± 6.0	14.9 ± 0.8
Muskogee	1	70.0 ± 1.0	20.1 ± 1.1	1.5 ± 5.4	8.4 ± 2.2
	5	61.4 ± 3.1	23.6 ± 0.8	1.6 ± 5.6	13.4 ± 2.8
	10	56.0 ± 0.6	25.5 ± 0.5	1.5 ± 4.8	16.9 ± 1.7
Chelyabinsk	1	37.6 ± 0.5	32.7 ± 1.2	1.8 ± 5.2	27.9 ± 3.1
	5	13.9 ± 0.2	46.4 ± 3.1	1.7 ± 3.0	37.2 ± 2.8
	10	11.9 ± 0.9	50.9 ± 1.8	0.2 ± 2.9	37.2 ± 3.3

**Table 4:** Adhesion values of HeLa cells after 24h incubation with fly ash samples measured with atomic force microscopy.

Control	Rockdale	Dolet Hills	Rockport	Muskogee	Chelyabinsk
9.4 ± 3.3	7.1 ± 2.9	11.2 ± 1.9	17.2 ± 4.4	12.5 ± 3.4	10.7 ± 2.0

## Data Availability

The data presented in this study are available on request from the corresponding author.

## Supplementary Materials

Figure S1. Flow cytometry data demonstrating the viability of Jurkat cells stained with Annexin V-FITC (apoptotic cells) and propidium iodide (dead cells) dyes. The data demonstrates the weak induction of early apoptosis and high induction of later apoptosis and necrosis in cells incubated with fly ash. Figure S2. AFM images of fly ash from Chelyabinsk. Cantilever approaching the fly ash sample (image obtained with the optical camera of the microscope) (A); surface topography of Chelyabinsk fly ash particles (B); non-specific adhesion of the fly ash particle surface (C).

## Sample Availability

Samples of the compounds are not available from the authors.
