# Supplementary material for: Comparative Toxicity of Fly Ash: An In Vitro Study
Source: Molecules. 2021 Mar 30;26(7):1926. doi: 10.3390/molecules26071926 (PMC8038091; doi:10.3390/molecules26071926)
Supplement: Supplementary file 1 [file molecules-26-01926-s001.pdf]

Supplementary Material

Comparative Toxicity of Fly Ash: An in Vitro Study

Elvira Rozhina [1], Ilnur Ishmukhametov [1], Läysän Nigamatzyanova [1], Farida Akhatova [1], Svetlana Batasheva [1], Sergey Taskaev [2], Carlos Montes [3], Yuri Lvov [3] and Rawil Fakhrullin [1,*]

[1] Bionanotechnology Lab, Institute of Fundamental Medicine and Biology, Kazan Federal University, Kreml uramı 18, Kazan, Republic of Tatarstan, 420008, Russian Federation

[2] Physics Departament, Chelyabinsk State University, 129 Bratiev Kashirinykh st., Chelyabinsk, 454001, Russian Federation

[3] Institute for Micromanufacturing, Louisiana Tech University, Ruston, LA 71272, USA

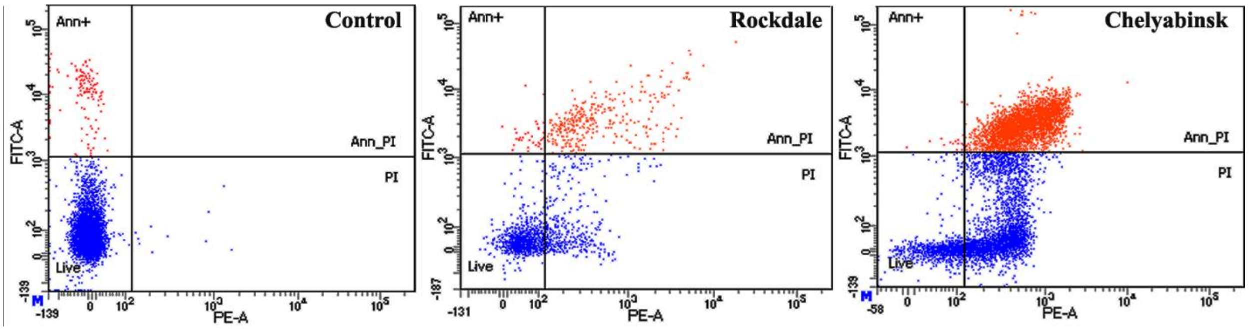

Figure S1. Flow cytometry data demonstrating the viability of Jurkat cells stained with Annexin V-FITC (apoptotic cells) and propidium iodide (dead cells) dyes. The data demonstrates the weak induction of early apoptosis and high induction of later apoptosis and necrosis in cells incubated with fly ash.

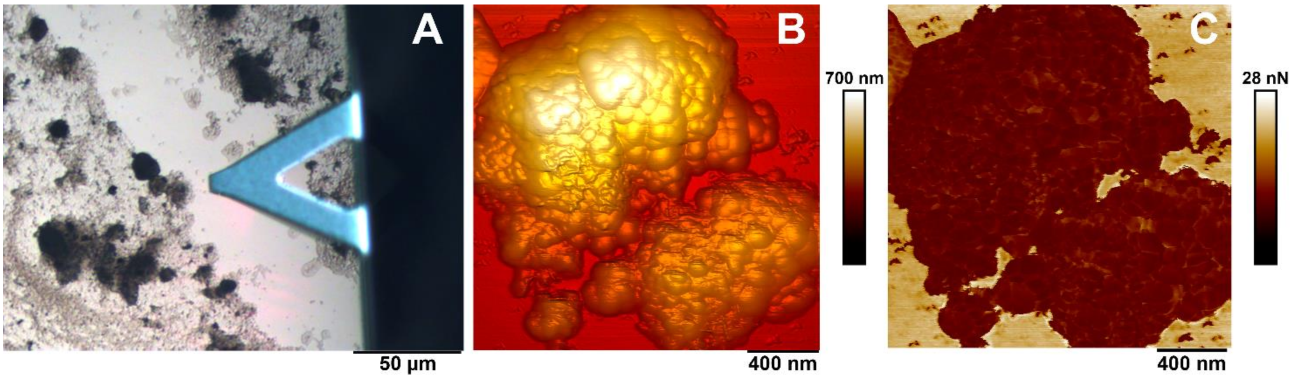

Figure S2. AFM images of fly ash from Chelyabinsk. Cantilever approaching the fly ash sample (image obtained with the optical camera of the microscope) (**A**); surface topography of Chelyabinsk fly ash particles (**B**); non-specific adhesion of the fly ash particle surface (**C**).
